# Dirty habits: potential for spread of antibiotic-resistance by black-headed gulls from waste-water treatment plants

**DOI:** 10.1007/s11356-024-35551-5

**Published:** 2024-11-30

**Authors:** Víctor Martín-Vélez, Joan Navarro, Manuel Vazquez, María J. Navarro-Ramos, Jonas Bonnedahl, Mariëlle L. van Toor, Javier Bustamante, Andy J. Green

**Affiliations:** 1 Institut de Ciències del Mar (ICM), CSIC, Passeig Marítim de la Barceloneta 37-49, 08003 Barcelona, Spain; 2 Departamento de Ciencias de La Vida, Universidad de Alcalá, Alcalá de Henares, Madrid Spain; 3 Department of Conservation Biology and Global Change, Estación Biológica de Doñana (EBD), CSIC, Américo Vespucio 26, 41092 Seville, Spain; 4 Department of Biomedical and Clinical Sciences, Linköping University, 581 83 Linköping, Sweden; 5 Department of Infectious Diseases, Region Kalmar County, 391 85 Kalmar, Sweden; 6 Centre for Ecology and Evolution in Microbial Model Systems, Linnaeus University, Stuvaregatan 2, 392 31 Kalmar, Sweden

**Keywords:** *Chroicocephalus ridibundus*, *Escherichia coli*, Landfills, GPS tracking, WWTPs

## Abstract

Anthropogenic environments such as wastewater treatment plants (WWTPs) and landfills are sources of antimicrobial resistance (AMR). Black-headed gulls (*Chroicocephalus ridibundus)* frequently use WWTPs and may be vectors for AMR. We used GPS tracking data for 39 gulls for up to 8 months, combined with a shedding curve, to study sources and dispersal distances of AMR in Iberia. The gulls used 21 different WWTPs (684 visits) and three landfills (21 visits). Areas of high risk of AMR dissemination were an average of 25 km from the infection source, with a maximum of 500 km. Solar saltworks and natural waterbodies were particularly exposed to AMR dissemination, followed by agriculture, sports facilities, and tourist beaches. There was important variability between individual gulls in their habitat specialization, and which WWTPs they visited. Studying the spatial movements of gulls after visiting WWTPs and landfills helps pinpoint sensitive locations where pathogen transmission is most likely.

**Supplementary Information:**

The online version contains supplementary material available at 10.1007/s11356-024-35551-5.

## Introduction

Antimicrobial resistance (AMR) is a growing threat to public human health, and a major global health challenge for this century (Ahmad and Khan 2019). AMR may undermine treatments against bacterial infections by the transference of resistance genes between humans, wildlife, and the environment (Alm et al. 2018). AMR genes can constitute a selective advantage in habitats and environments with a high load of antibiotic residues, biocides, or heavy metals due to anthropogenic inputs (Gullberg et al. 2014). Waste water treatment plants (WWTPs) and landfills are particularly important hotspots for AMR proliferation and horizontal gene transfer (Pallares-Vega et al. 2019; Wu et al. 2017). Although the sources of AMR have been studied in depth, the pathways of spread in the environment are less understood (Swift et al. 2019). Wildlife using anthropogenic habitats can be important vectors in the emergence, circulation, and spread of pathogens, including antibiotic-resistant bacteria (Cunningham et al. 2017). However, few studies have addressed the spatial patterns of AMR dissemination by wildlife, and the areas that are affected (Dolejska 2020; Ahlstrom et al. 2021; Martín-Vélez et al. 2024a).

Gulls are opportunistic feeders that exploit anthropogenic environments, and small-sized species such as the black-headed gulls (*Chroicocephalus ridibundus*) frequently use WWTPs and landfills (Arnold et al. 2016; Nguyen et al. 2021). Gulls can be infected by AMR in WWTPs by feeding on human fecal material at sewage works or their outflows (Ferns and Mudge 2000; Clark et al. 2013; Woksepp et al. 2023), and infection in landfills is expected while feeding in waste and lixiviates (Sacristán-Soriano et al. 2024).

Black-headed gulls can be important reservoirs and hosts of AMR (Velhner et al. 2021; Woksepp et al. 2023) and act as vectors for long-distance pathogen dispersal (Gan et al. 2019). The black-headed gull is a migratory and common breeder across Western Europe, with many large colonies in France, UK, Belgium, and Italy (Wetlands International 2023). It is also abundant during both breeding and wintering seasons in the Iberian Peninsula, with around 170,000 in Spain and 69,000 in Portugal (https://ebird.org/species/bkhgul/ES).

The development of GPS devices allows for the tracking of black-headed gull movements (Jakubas et al. 2020; Indykiewicz et al. 2021), including visits to sources of AMR, and to sensitive habitats potentially affected by AMR dissemination by these gulls. Spread of AMR by black-headed gulls, which include Enterobacterales such as *Escherichia coli*, *Enterobacter* sp., *Raoultella ornithinolytica*, and *Klebsiella pneumoniae*, is expected to occur through fecal contamination (Velhner et al. 2021; Woksepp et al. 2023). Antimicrobial resistance may contaminate surface waters used for irrigation, drinking, recreation (Reed et al. 2003), food production (e.g., agriculture, solar salt works or “salines”; Moré et al. 2017), and areas with high human densities (e.g., beaches, urban parks, sport facilities), which increases the probability of pathogen dispersal to people (Ngaiganam et al. 2019; Woksepp et al. 2023). The extent of AMR dissemination may also depend on specialization by individual gulls. For example, perhaps only a subset of individuals use WWTPs, or perhaps generalist individuals in the population would disseminate AMR through a wider range of potential AMR recipient areas than specialized individuals (Navarro et al. 2017).

Our objective was to investigate the expected spatial patterns of AMR dispersal by black-headed gulls in Southern Iberia. We integrated the movements collected from GPS-tracked black-headed gulls with spatial land use information to identify the likely sources and recipient areas for AMR dissemination. Specifically, we aimed to (1) identify major sources of AMR (WWTPs and landfills) used by black-headed gulls in the southern Iberian Peninsula, and determine their relative importance; (2) estimate AMR dissemination distances from these sources, and map spatial probabilities of AMR dispersal; (3) identify land-use types that may be particularly affected by AMR dissemination, and (4) examine the degree of individual specialization in habitat use, and hence differences in AMR dispersal potential.

## Methods

### Fieldwork procedures and data management

**Fig. 1:**
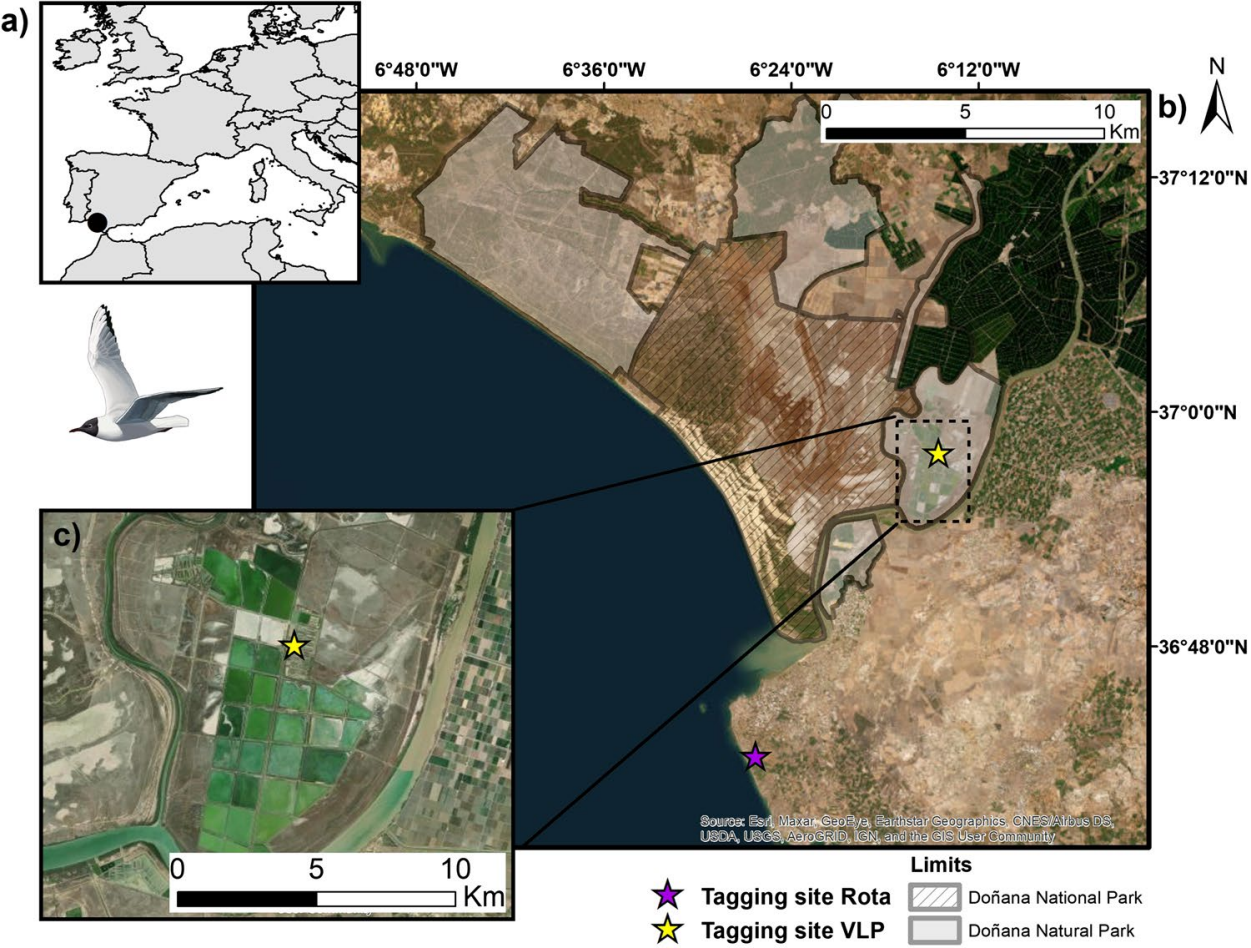
**a** Location of capture areas within Spain and Europe. **b** Location of capture sites at Veta la Palma fish ponds within Doñana, and in Costa Ballena in Rota, Cadiz. **c** Location of the black-headed gull tagging site within VLP. Image credits: Martí Franch.

We deployed GPS/GSM transmitters on 39 black-headed gull individuals during the breeding season (April-June) from a breeding colony of 700 pairs in 2022 in Veta la Palma (VLP) (Sevilla province, south-western Spain), and during the wintering season (November 2022) in VLP and in Costa Ballena (Rota, Cádiz province, Spain) (Fig. 1). VLP is located within Doñana Natural Park (Fig. 1a), bordered by Doñana National Park to the west and the Guadalquivir estuary to the east and south (Fig. 1b). VLP was created in the 1990s from the conversion of 3000 ha of seasonal marshes into 40 large shallow fish ponds (Fig. 1c). VLP lies within a Ramsar Site and Important Bird Area with records of around 250 species (Walton et al. 2015; Green et al. 2018). These fishponds are important habitats for waterbirds throughout the year (Rendón et al. 2008; Sebastián-González and Green 2017). Costa Ballena tagging site is an urban park by the coast, which includes the outlet from a WWTP to an artificial lake.

Out of the 39 black-headed gulls, 32 adults were captured with a clap-net (ECOTONE, Poland) placed outside the breeding colony and baited with fish pellets in VLP (see Fig. 1c), and seven adults were captured in Costa Ballena with a cannon-net baited with bread (Fig. 1b). All gulls were deployed with similar GPS devices (Druid Technology Co, OMNI-2G loggers; Table S1). GPS units were set up with a 10-min frequency with the boost function activated. Boost provides more GPS positions (every 1 min) only if GPS devices have a full battery; otherwise, the frequency is set to 10 min. These are GPS-GSM devices that transmit data twice per day (set by default at 10:00 h and 18:00 h) to the database. All GPS devices were deployed using a Backpack Teflon ribbon harness (the GPS unit plus the harness weighed around 8 g), and thus less than the 3% body mass threshold suggested for gulls (Martín-Vélez et al. 2022; Passos et al. 2010). For the present study, we considered movements taking place from the tagging date until 31th January 2023.

### Potential pathogen spread risk maps

**Fig. 2:**
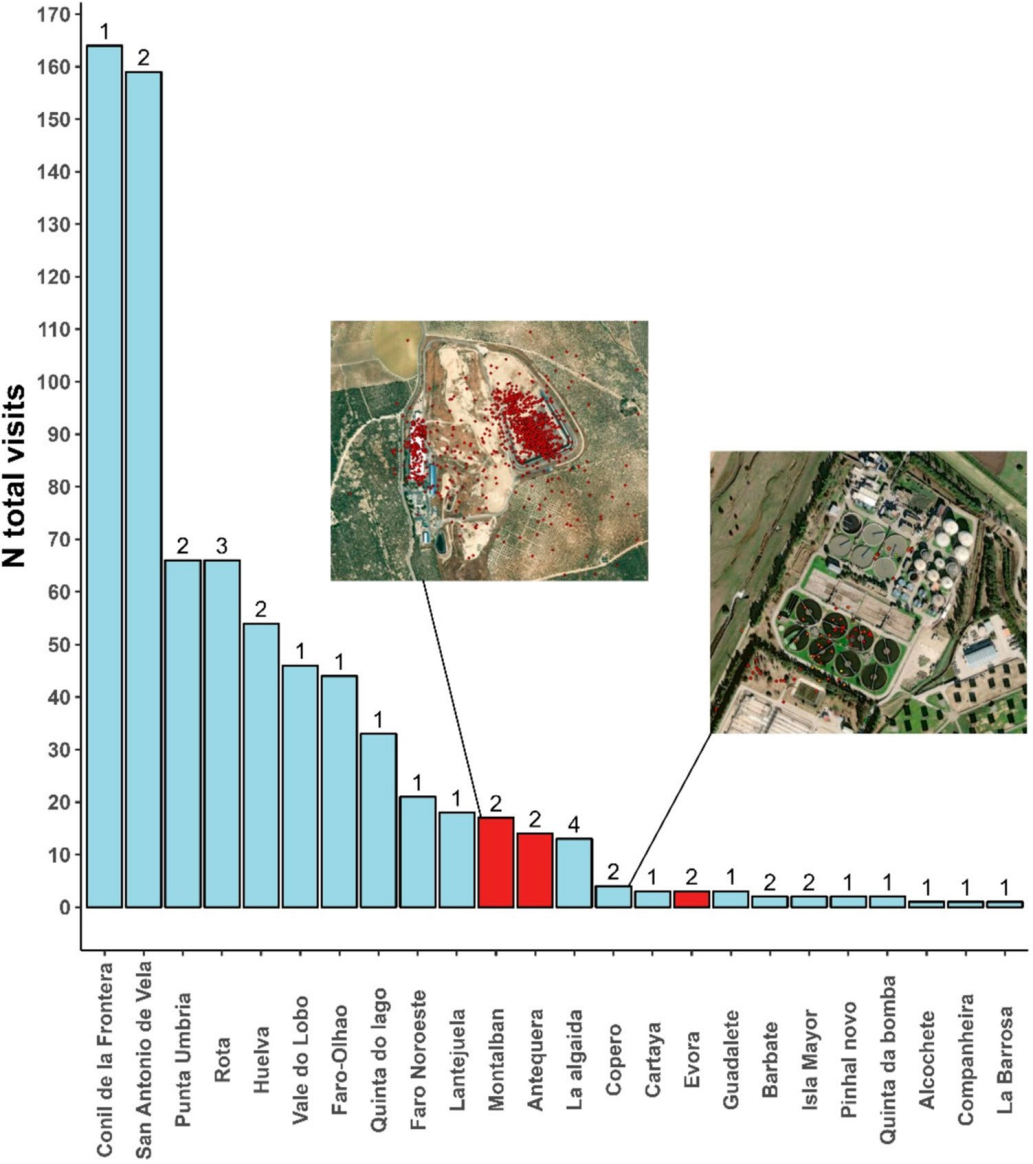
Number of visits made by tagged gulls to each of the WWTPs (in blue) or landfills (in red) visited during the study. The number of tagged gull individuals that visited each AMR source is given above each bar. Examples of a WWTP (e.g., Copero in Sevilla) and a landfill (Montalban, Cordoba) used are given with satellite images

We considered all WWTPs and landfills that were actively used by the black-headed gulls within the Iberian Peninsula as point sources for AMR. We did not screen the black-headed gulls we tracked for antibiotic-resistant bacteria (ARBs) or antibiotic-resistance genes (ARGs) and assumed that AMR concentrations in individuals likely depend on the degree of exposure to AMR sources (see Woksepp et al. 2023 for supporting evidence). Some individuals moved to Africa, but we did not include movements there owing to greater difficulty in identifying AMR sources. We plotted the GPS fixes of the individual gulls in Google Earth and located WWTPs and landfills potentially used by the gulls. We identified a WWTP based on visual training while searching for circular ponds present in the treatment plants (see Fig. 2). We applied a 200-m buffer to the WWTP centroid coordinates and overlapped the polygons with the GPS data. We selected landfills based on the EUROPEAN layer for waste management (https://ec.europa.eu/eurostat/web/waste/data/database). Points were georeferenced to the centroid of the dump under visual inspection (Fig. 2), and a 200-m buffer was applied. We selected GPS points with a speed lower than 3 m s^−1^ over the WWTP/landfill as an indicator that the individual was actively using the area (Martín-Vélez et al. 2020; López-Calderón et al. 2023). For each WWTP and landfill used by gulls that we identified, we calculated the total number of visits per individual (see below), and the total number of visiting individuals.

The shedding time of AMR carriage for resistant *Escherichia coli* in waterbirds varies considerably (Dolejska 2020). Experimental evidence in ring-billed gulls (*Larus delawarensis*) suggests that AMR concentration (Colistin-resistance *E. coli*- log_10_ Colony Formation Unit [CFU]/g) for *E. coli* follows a lognormal curve during the first 30 days (Franklin et al. 2020; Ahlstrom et al. 2021; Figure S1). Therefore, we selected the WWTPs/landfills as starting points of each GPS trajectory (*t*_*0*_) and selected the GPS points within the next 30 days after the individual departed. We considered that a visit was made to an AMR source when an individual gull was over that source with instantaneous speed < 3 m s^−1^ on a given date (gull-days). We selected GPS points every 2.37 h from the departure (*t*_*0*_), as this is the defecation rate expected for black-headed gulls (Hahn et al. 2007). We then associated a daily concentration (Colistin-resistance *E. coli*-log_10_ CFU/g) derived from a lognormal curve generated by Franklin et al. (2020) to the GPS fixes for defecation events after the departure from the AMR source (Figure S1). We used the mean estimates as well as the lower and upper estimates of a 95% confidence interval of the lognormal shedding model to account for uncertainty in the shedding duration (Figure S1). When gulls visited an AMR source (i.e., a WWTP or a landfill) repeatedly over several days, we assigned a weight to the subsequent days according to how many visits occurred during the complete shedding period of 30 days. Maximum dispersal distances (Haversine distance from the first GPS point of the trajectory at *t*_*0*_) from the AMR source to the point of defecation were calculated for each trajectory departing from a given source.

We generated a spatially explicit risk map for AMR spread in the Iberian Peninsula (Portugal and Spain) considering a 500-m neighboring area of influence around each GPS egestion point. We used the *Inverse Distance Weight* (IDW) tool from ArcMap 10.8 with a 100-m resolution. Points were weighted based on a correction value derived from the Colony Formation Unit (CFU) concentration, itself extracted from the shedding time curve (Figure S1) and the number of visits to a single AMR source. After generating the IDW raster, external contours (with 100% of the data) of the total area potentially affected by gulls were extracted (based on the 500 m distance determined for IDW), and the total area was calculated. The final visualization of the high-probability transmission areas was performed in *ArcMap 10.8*.

### Recipient areas used by gulls, and individual specialization

We overlapped the GPS egestion points of the trajectories departing from WWTPs and landfills (AMR sources) with the European Land Use layer CORINE 2018 (Coordination of Information on the Environment, CLC; https://land.copernicus.eu/) common to both Portugal and Spain. We reclassified the CORINE categories into 11 main categories (Table S2): (1) beach, (2) forest, (3) industrial, (4) green urban areas (5) other urban, (6) ricefields, (7) other agriculture types (e.g., non-irrigated arable lands, permanently irrigated land, vineyards, olive trees, fruit trees, and annual crops), (8) natural waterbodies, (9) ports, (10) solar saltworks (salines) and (11) sport facilities. We specifically considered the effect of gulls on beaches, green urban areas, and sports facilities (e.g., golf courses) as land uses with high aggregations of human population and thus higher probability of pathogen dissemination (Alm et al. 2018; Ngaiganam et al. 2019). Industrial, rice fields, ports, and solar saltworks are also considered potentially sensitive for AMR dispersal (Navarro et al. 2019; Firth et al. 2020).

For those individuals who visited WWTPs or landfills, we then calculated an individual specialization index for land use following Bolnick et al. (2003) and Fodrie et al. (2015). We calculated the number of visits to different AMR sources by each tagged individual, and the percentage of land use of each individual black-headed gull after visiting the WWTPs/landfills. We also calculated the average specialization index for all tracked individuals (i.e., all the tagged population). Specifically, we calculated the proportional land use by each individual gull (and for the whole set of marked individuals) as the number of GPS points in a particular habitat divided by the total number of GPS positions recorded for that individual within 30 days of departing a WWTP/landfill. We calculated the Proportional Similarity Index (PSi) using the proportion of GPS positions in each habitat based on the R package *RinSp* (Zaccarelli et al. 2013). PSi measures the individual specialization based on habitat-by-habitat deviations in an individual’s habitat use relative to the population level (0 = more specialized; 1 = more generalist). Mean Psi (and error mean) among individuals was used to determine the average amount, or prevalence, of individual specialization in habitat use in the population. We ran Monte Carlo permutations (999 permutations) to test whether observed PSi values for individuals visiting WWTPs or landfills differed significantly from a random distribution of values subsampled from the whole population.

## Results

### Utilization of WWTPs and landfills by GPS-tracked individuals

**Fig. 3:**
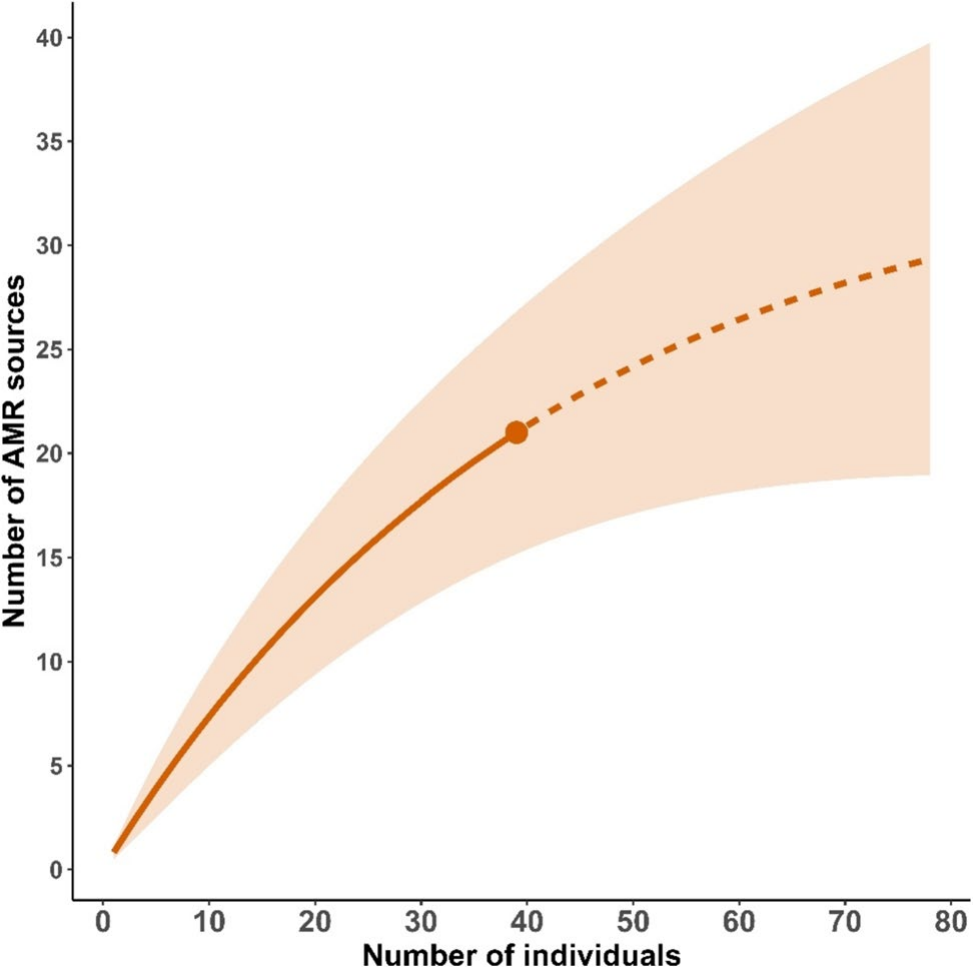
Rarefaction analysis showing the accumulated number of AMR sources (WWTPs and landfills) in relation to a number of tagged black-headed gulls (continuous line). The discontinuous line shows the extrapolation if the number of tagged individuals was increased. Error bars represent 95% confidence intervals

Eighteen tagged individual gulls visited at least one WWTP or landfill in the Iberian Peninsula during the tracking period (Table S1). From the 39 tagged individuals, we lost the signal for 5 of them (due to device failure or death) within the first 20 days of tracking, and 7 departed the Iberian Peninsula to winter in Africa (Morocco, Mauritania, Senegal) for wintering (mean time spent in Africa = 131 days; we lacked information on AMR sources there). Therefore, of 27 individuals who remained in the Iberian Peninsula, 21 (77%) visited at least one AMR source. A total of 21 WWTPs and 3 landfills divided between Portugal (9 WWTPs and 1 landfill) and Spain (12 WWTPs and 2 landfills) were visited by these 21 gulls over the 7-month study period (Table 1). Rarefaction analyses showed that, as the number of tagged gulls increased, the number of AMR sources visited continued to increase without reaching an asymptote (Fig. 3). Gulls performed 739 independent visits (gull-days) to AMR sources: 80 visits were performed by 6 of the 7 individuals tagged in Rota, and 659 gull-day visits were performed by 15 of the 32 individuals tagged in VLP.

**Table 1:** List of the waste water treatment plants (WWTPs) and Landfills used by the tagged black-headed gulls

Country	Provinces/districts	Name	Type	Latitude	Longitude	Plant size (p.e.)
Spain	Cadiz	Rota	WWTP	36.67	− 6.39	162,500
Spain	Cadiz	Barbate	WWTP	36.18	− 5.91	34,240
Spain	Cadiz	Conil de la frontera	WWTP	36.28	− 6.08	48,000
Spain	Cadiz	La Barrosa	WWTP	36.38	− 6.18	86,250
Spain	Cadiz	La Algaida	WWTP	36.79	− 6.30	103,000
Spain	Cadiz	Guadalete	WWTP	36.64	− 6.12	864,000
Spain	Huelva	Huelva	WWTP	37.24	− 6.93	311,400
Spain	Huelva	Punta Umbria	WWTP	37.21	− 7.01	142,000
Spain	Huelva	Cartaya	WWTP	37.28	− 7.17	57,192
Spain	Sevilla	Copero	WWTP	37.31	− 5.99	950,000
Spain	Sevilla	Isla Mayor	WWTP	37.13	− 6.16	10,000
Spain	Sevilla	Lantejuela	WWTP	37.36	− 5.23	5,200
Portugal	Faro	San Antonio de Vela	WWTP	37.21	− 7.42	116,500
Portugal	Faro	Faro-Olhao	WWTP	37.02	− 7.90	113,200
Portugal	Faro	Faro Noroeste	WWTP	37.02	− 7.96	44,530
Portugal	Faro	Vale do lobo	WWTP	37.06	− 8.07	8100
Portugal	Faro	Quinta do lago	WWTP	37.04	− 8.10	27,000
Portugal	Faro	Companheira	WWTP	37.15	− 8.52	140,092
Portugal	Lisbon	Quinta da bomba	WWTP	38.65	− 9.14	198,290
Portugal	Lisbon	Pinhal novo	WWTP	38.65	− 8.88	24,000
Portugal	Lisbon	Alcochete	WWTP	38.74	− 8.97	27,750
Country	Province/districts	Name	Type	Latitude	Longitude	Waste (tons/year)
Spain	Malaga	Antequera	Dump	37.02	− 4.64	294,912
Spain	Cordoba	Montalban	Dump	37.51	− 4.76	85,603
Portugal	Evora	Evora	Dump	38.54	− 7.97	–

Country, province, name, coordinates, and season are provided. Plant size (population equivalent (p.e.)) is provided for WWTP and Waste generated (in tons per year) for landfills as indicators of the carrying capacity of each site. Source for plant size in WWTP: https://water.europa.eu/freshwater/countries/uwwt/spain

Source for waste generated in landfills: Plan Integral de Residuos de Andalucía 2021

WWTPs were visited more often than landfills, receiving 95.5% of all visits made to AMR sources (Fig. 2). The sites visited by most individuals were La Barrosa WWTP with four individuals and Rota WWTP with three individuals (both in Cadiz province, south-west Spain, Fig. 2). The maximum number of day visits to any AMR source was made to the Conil de la Frontera WWTP (Spain, 164 gull-days from one individual) and San Vicente de Vila WWTP (Portugal, 154 gull-days divided between two individuals) (Fig. 2).

### Risk map for AMR spread

**Fig. 4:**
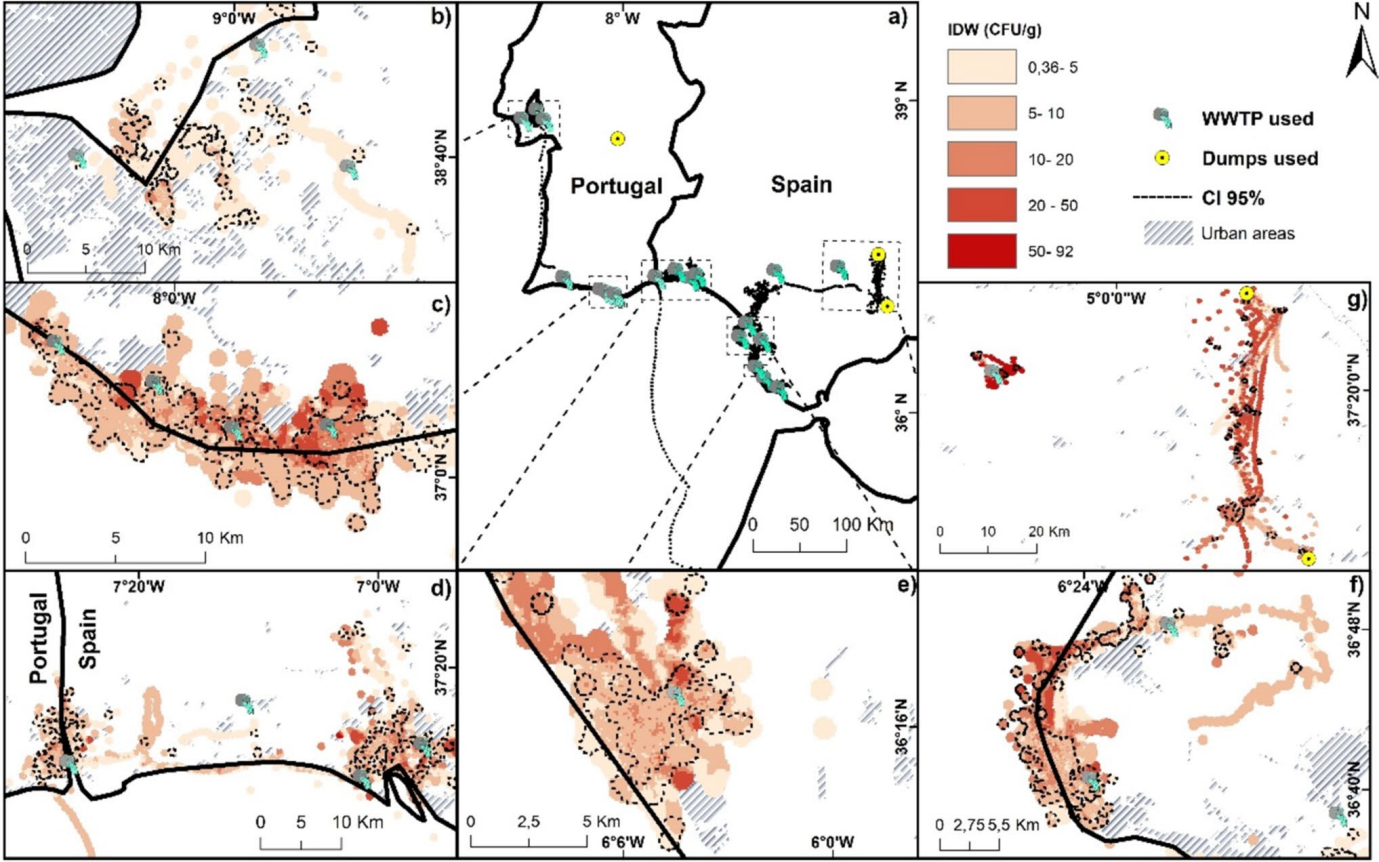
**a** Location of waste water treatment plants (WWTPs, blue dots) and landfills (yellow dots) used by black-headed gulls in the Iberian Peninsula. Close-ups show the concentrations of potential AMR dissemination (CFU/g) based on Inverse Distance Weight (IDW) showing the main zones for high risk transmission in **b** surroundings of Lisbon, **c** Faro, **d** boundary between Portugal (Vila Real de San Antonio) and Spain (Huelva), **e** Conil de la Frontera (Cadiz), **f** Rota (Cadiz) and **g** Lantejuela (Sevilla, on the left side) and Fuente de Piedra (Malaga, on the right side). 95% confidence intervals (CIs) for affected areas were based on the shedding curve from Franklin et al. (2020)

Gull movements within the 30 days after the visit of a WWTP or a landfill were concentrated along the Portuguese coast near Lisbon and Faro (Fig. 4b, c), and along the southern Spanish coast of Huelva and Cadiz provinces (Fig. 4d–f) covering a Minimum Convex Polygon area of 65,000 km^2^. Furthermore, we identified some inland hotspots in Sevilla and Malaga provinces (Fig. 4g). Overall, the maximum potential area with a risk of AMR transmission was 538.58 km^2^ (with CI 95% ranging from 295 to 561 km^2^) (Fig. 4a). However, areas of highest probability of transmission were concentrated mainly in Faro (Fig. 4c), San Vicente de Vila (Fig. 4d), Huelva (including Odiel marshes; Fig. 4d) and some specific sites in Cadiz (Rota and Conil de la Frontera; Fig. 4e, f). The median transmission distance from the source of AMR to recipient habitats was 24.18 km (arithmetic mean of 78.28 km), with a maximum distance of 524 km (Figure S2).

### Recipient area sensitivity and individual specialization

**Fig. 5:**
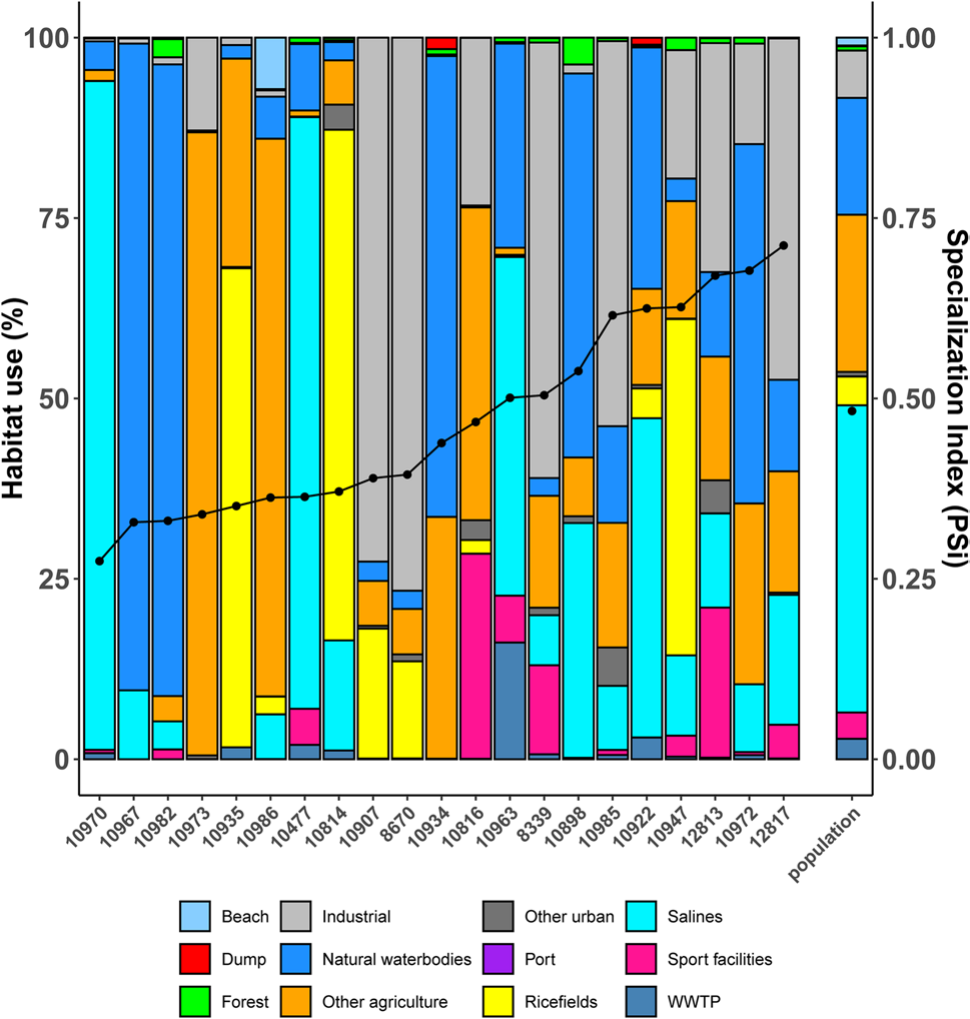
Individual variation in habitats used during the 30-day interval after visiting AMR sources for 21 GPS-tracked black-headed gulls from May 2022 to January 2023. Each individual (*x*-axis) is represented by a vertical bar (with the individual identifier), subdivided by the proportion of locations in each habitat in relation to the total number of GPS locations. The habitat specialization index PSi is indicated by black dots (connected with a black line; 0 = extreme specialist; 1 = extreme generalist). Mean habitat use and mean PSi for the set of tagged individuals that visited an AMR source are given in the right-hand bar

We detected the potential impact of AMR dissemination, after visits to WWTPs or landfills, in ten land-use types (Fig. 5). At the population level, the relative impact on each type (as a percentage of total locations over the 30-day interval after visits to WWTPs or landfills) ranged from 43 and 19% (for saltworks and natural waterbodies, respectively) to less than 1% (green urban areas, beaches, and forests; see Fig. 5). “Other agriculture” (i.e., excluding rice fields) scored around 16% of the total surface area used after visiting AMR sources (Fig. 5). At the individual level, the mean PSi value was 0.48 ± 0.03 s.e. and ranged from 0.17 to 0.72 (values close to 0 indicate specialists, those close to 1 indicate generalists). Specialist individuals focused on saltworks, natural waterbodies, and “other agriculture” (Fig. 5). Monte Carlo analyses of individual-versus-population niche variation indicated that specialized individuals were significantly prevalent within the set of birds that visited at least one WWTP or landfill (*p* < 0.001; Fig. 5).

## Discussion

Using detailed GPS movement data, we found that the widespread black-headed gull is an important user of WWTPs in Europe, with major implications for the dispersal of AMR. Even birds tagged within a small area of Spain visited 21 different WWTPs within an area of 65,000 km^2^, with occasional visits to three landfills as further sources of AMR, and potential visits to additional AMR sources in Africa. Along the Iberian coast, we estimated birds had median dissemination distances of 24 km from WWTPs or landfills, during a shedding time for resistant *E. coli* of 30 days. CORINE habitats of saltworks and natural waterbodies were the most exposed to gull AMR dissemination, but other potential recipient areas with more risk to humans were regularly exposed. There was major variability between gull individuals in their land use specialization after visiting AMR sources, as well as the identity of the WWTPs they visited. Our results highlight the potential of black-headed gulls as vectors of AMR and pathogens from WWTPs, and the importance of understanding spatially explicit processes of bacterial dissemination.

### Sources of AMR in the Iberian Peninsula

High concentrations of pathogens occur in WWTPs and landfills (Wu et al. 2017; Hiller et al. 2019). Wastewater (sewage) provides a continuous input of highly diverse commensal and pathogenic bacteria from human and animal microbiomes into WWTPs, acting as a reservoir of AMR (Taylor et al. 2011; Nguyen et al. 2021; Woksepp et al. 2023). Even after treatment, WWTP effluents flowing into rivers may be important sources of AMR and other pathogens. Antibiotic-resistant *Salmonella* strains were reported in WWTP effluents and black-headed gulls in the Czech Republic (Masarikova et al. 2016). In Sweden, antibiotic-resistant *Enterobacter* and *Raoultella* strains and ARGs were shared between a WWTP and black-headed gull feces from there or a nearby park (Woksepp et al. 2023). High concentrations of antibiotics and heavy metals in landfills also enhance AMR proliferation (Wu et al. 2017). Landfills produce leachates with high AMR concentrations that contaminate surroundings (Scott et al. 2005; Jarma et al. 2024). Gulls and storks that use landfills in Spain carry more AMR than other sympatric waterbirds that avoid landfills (Jarma et al. 2021).

### Use of AMR sources by black-headed gulls

Black-headed gulls regularly use WWTPs throughout the non-breeding season in the Iberian Peninsula and elsewhere in Europe (Carroll et al. 2015). This is related to the small size of the black-headed gull and its association with freshwater (Gough et al. 2003; Fernie and Letcher 2018). Our results contrast with those for larger gulls (yellow-legged gull *Larus michahellis*, and Lesser black-backed gull *Larus fuscus*) in south-west Spain, which make intensive use of many landfills but not of WWTPs (Navarro et al. 2019; Martín-Vélez et al. 2024b, 2020; Ramírez et al. 2020). In contrast, the medium-size slender-billed gull *Larus genei* is common in saltworks, but is not reported from WWTPs or landfills (Ramírez et al. 2012). White-storks *Ciconia ciconia* are also abundant at landfills (but not WWTPs) in the study region (López-Calderón et al. 2023).

Although 31 of the tagged gulls were active breeders in VLP, 18 WWTPs used by them were located along the Iberian coast from Lisbon and surroundings (> 300 km from the breeding colony), through southern Portugal (Faro and Algarve) to southern Spain (Huelva, Cadiz). The selection of coastal WWTPs may be related to the use by black-headed gulls in coastal marshlands and saltworks (Kubetzki and Garthe 2003). However, these generalist foragers are also common in inland wetlands and anthropogenic habitats, including urban parks and landfills (Velhner et al. 2021; Woksepp et al. 2023). We detected three landfills used by GPS-tracked individuals: one in Evora, Portugal, and two near Fuente de Piedra Lake in Malaga (Antequera and Montalban, see also Martín-Vélez et al. 2024b, Jarma et al. 2024). It remains unclear what characteristics of WWTPs or landfills may increase their attractiveness to the species. Specific interventions (e.g., preventing gulls from accessing sewage exposed in sedimentation ponds) at WWTPs might help to reduce the spread of AMR (Clark et al. 2013; Huijbers et al. 2015; Woksepp et al. 2023). Concentrations of AMR at WWTPs can be reduced with additional treatment (e.g., ozone, UV, ultrafiltration, Goldstein et al. 2014).

### Areas at risk from AMR dispersal by gulls

Black-headed gulls used ten different land-use types potentially affected by AMR dispersal. Aquatic environments (saltworks, and natural waterbodies) were the most important habitats for AMR dispersal by gulls. Contamination of hypersaline habitats would be of least concern, as salinity attenuates the effects of AMR (Zhang et al. 2019). However, many sites classified by CORINE land cover as “saltworks” are in fact aquaculture farms (e.g., VLP, Esteros del Guadalquivir near Doñana and Acuinova in Ayamonte, Huelva), which are not hypersaline, and in which AMR dispersal poses risks for commercial fish production and public health (Berg and Anderson 1972). Agricultural landscapes were affected by AMR dispersal, with implications for food production (Thanner et al. 2016). Finally, habitats and/or installations with high densities of people (e.g., sport facilities, which were usually golf courses) would increase the probability of AMR dispersal, especially for sensitive fractions of the population such as the elderly (Mellata 2013; Ngaiganam et al. 2019).

### Individual habitat specialization associated with AMR spread

The individual specialization index showed that some gulls were specialized in the use of particular environments. Individuals who visit AMR sources and also specialize in using sensitive land use types (e.g., those used for food production) may be of special concern due to accumulation effects (Devarajan et al. 2015). In addition, we weighted the probability of AMR transmission based on the consecutive number of visits. Therefore, sources that accumulate multiple visits by an individual would have higher probabilities of AMR contamination and potential dissemination to humans or livestock (Ahlstrom et al. 2018). The true effect of gull dispersal in the environment is difficult to quantify, as the fate of AMR shed into different habitats is unknown. Furthermore, shedding times of infection are variable among bird species, pathogen species (and strains), individual birds, seasons, and locations (Swift et al. 2019; Zeballos-Gross et al. 2021), and more experimental research is required to understand this variation.

We used a conservative approach to filter a threshold for 30 days, yet individuals infected with ARBs may still shed them after > 30 days (Figure S1; Franklin et al. 2020; Ahlstrom et al. 2021). We did not screen the black-headed gulls we tracked for AMR, and concentrations in individuals likely depend on the degree of exposure to AMR sources, as well as potential variation in immune response. Future studies that include the testing of monitored black-headed gulls for AMRs, and confirm whether individual concentrations depend on the degree of exposure to AMR would be recommended.”

### Implications for AMR dispersal by black-headed gulls

Half of the AMR dissemination we estimated was within a 24-km radius of the WWTP or landfill, with a maximum of up to 500 km (to Morocco) with a much lower probability of AMR dispersal (Figure S2). Other gull studies with different methodologies reported a wide range of AMR dispersal distances: from 5 to 30 km in yellow-legged gulls (Navarro et al. 2019), and up to 3000 km for herring gulls *Larus argentatus* during migration (Ahlstrom et al. 2021). In our case, potentially infected black-headed gulls often moved between Spain and Portugal (see Fig. 1a) stressing the importance of inter-country health regulations (e.g., EU directives for waste management in landfills, and wastewater treatment) and cooperation in disease control (Navarro et al. 2019). Furthermore, non-breeding black-headed gulls tagged while wintering in Spain traveled to Northern Europe for breeding (data not shown), potentially dispersing AMR along their migration routes.

Black-headed gulls are one of the most abundant gull species and are ubiquitous in Eurasia (Wetlands International 2023). In the Iberian Peninsula, 170,000 wintering individuals were counted in 2021 (https://ebird.org/species/bkhgul/ES). Thousands of WWTPs are present across the Iberian Peninsula and other European countries. Given their abundance and widespread distribution, the role of black-headed gulls as potential vectors of AMR from WWTPs into other areas is of considerable importance. Studying the spatial patterns of movement of these gulls at wider scales, whilst screening gulls for ARB and ARG concentrations, would help to pinpoint sensitive locations where AMR transmission may occur so that risk reduction measures can be implemented.

## Supplementary Information

Below is the link to the electronic supplementary material.

Supplementary file1 (DOCX 208 KB)

## Data Availability

Data will be available at the CSIC repository.
