# Supplementary material for: Dirty habits: potential for spread of antibiotic-resistance by black-headed gulls from waste-water treatment plants
Source: Environ Sci Pollut Res Int. 2024 Nov 30;31(58):66079–89. doi: 10.1007/s11356-024-35551-5 (PMC11659338; doi:10.1007/s11356-024-35551-5)
Supplement: Supplementary file 1 — Supplementary file1 (DOCX 208 KB) [file 11356_2024_35551_MOESM1_ESM.docx]

**Supplementary Information**

Title: *Dirty habits: potential for spread of antibiotic-resistance by black-headed gulls from waste-water treatment plants*

**Table S1.** Summary of the data included in the analyses from 39 gull individuals tagged (30 in Veta la Palma and 9 in Rota during 2022), showing data up until 31^th^ January 2023. Individual gull identification (ID), date of the first and last GPS location, total duration of the track and number of visited AMR sources are shown.

| ID | Date of the first GPS location | Date of the last GPS location | Total duration (days) | AMR sources visited |
| --- | --- | --- | --- | --- |
| Veta la palma | | | |  |
| 10972 | 17/05/2022 | Still transmitting | - | 4 |
| 10967 | 07/06/2022 | Still transmitting | - | 1 |
| 10965 | 17/05/2022 | 20/07/2022 | 64 | 0 |
| 10927 | 11/05/2022 | 30/05/2022 | 19 | 0 |
| 10927_1 | 07/06/2022 | 01/09/2022 | 86 | 0 |
| 10898 | 11/05/2022 | Still transmitting | - | 2 |
| 10977 | 13/05/2022 | Still transmitting | - | 0 |
| 10986 | 25/05/2022 | Still transmitting | - | 3 |
| 10964 | 27/05/2022 | Still transmitting | - | 0 |
| 10938 | 24/05/2022 | 18/08/2022 | 86 | 0 |
| 10932 | 27/05/2022 | Still transmitting | - | 0 |
| 10478 | 07/06/2022 | Still transmitting | - | 0 |
| 10970 | 17/05/2022 | Still transmitting | - | 1 |
| 10906 | 07/06/2022 | 11/08/2022 | 65 | 0 |
| 10981 | 01/06/2022 | Still transmitting | - | 0 |
| 10963 | 31/05/2022 | Still transmitting | - | 4 |
| 10934 | 24/05/2022 | Still transmitting | - | 1 |
| 10969 | 25/05/2022 | 28/05/2022 | 3 | 0 |
| 10982 | 25/05/2022 | Still transmitting | - | 2 |
| 10973 | 24/05/2022 | 08/06/2022 | 15 | 1 |
| 10477 | 31/05/2022 | Still transmitting | - | 1 |
| 10904 | 31/05/2022 | 31/05/2022 | 1 | 0 |
| 10921 | 31/05/2022 | 17/09/2022 | 109 | 1 |
| 10984 | 27/05/2022 | Still transmitting | - | 0 |
| 10909 | 24/05/2022 | 30/05/2022 | 6 | 0 |
| 10468 | 01/06/2022 | Still transmitting | - | 0 |
| 10922 | 31/05/2022 | Still transmitting | - | 4 |
| 10945 | 17/05/2022 | Still transmitting | - | 0 |
| 10935 | 24/05/2022 | Still transmitting | - | 1 |
| 10814 | 27/05/2022 | Still transmitting | - | 2 |
| Rota | | | |  |
| 10947 | 10/11/2022 | Still transmitting | - | 2 |
| 10973_1 | 10/11/2022 | 14/11/2022 | 4 | 0 |
| 8670 | 21/11/2022 | 19/12/2022 | 28 | 1 |
| 10907 | 21/11/2022 | Still transmitting | - | 0 |
| 10985 | 28/11/2022 | Still transmitting | - | 1 |
| 8339 | 28/11/2022 | Still transmitting | - | 1 |
| 12813 | 28/11/2022 | Still transmitting | - | 2 |
| 12817 | 28/11/2022 | Still transmitting | - | 1 |
| 10816 | 28/11/2022 | Still transmitting | - | 1 |

**Table S2.** Reclassification from CORINE Land Cover 2018 for 11 categories used in our study that are potentially exposed to AMR dissemination.

| Corine classification | Reclassification |
| --- | --- |
| Beaches, dunes, sands | Beach |
| Dump sites | Dump |
| Broad-leaved forest | Forest |
| Coniferous forest | Forest |
| Mixed forest | Forest |
| Natural grasslands | Forest |
| Moors and heathland | Forest |
| Sclerophyllous vegetation | Forest |
| Transitional woodland-shrub | Forest |
| Bare rocks | Forest |
| Sparsely vegetated areas | Forest |
| Burnt areas | Forest |
| Green urban areas | Green Urban Areas |
| Continuous urban fabric | Industrial |
| Discontinuous urban fabric | Industrial |
| Industrial or commercial units | Industrial |
| Inland marshes | Natural waterbodies |
| Peat bogs | Natural waterbodies |
| Salt marshes | Natural waterbodies |
| Intertidal flats | Natural waterbodies |
| Water courses | Natural waterbodies |
| Water bodies | Natural waterbodies |
| Coastal lagoons | Natural waterbodies |
| Estuaries | Natural waterbodies |
| Sea and ocean | Natural waterbodies |
| Non-irrigated arable land | Other agriculture |
| Permanently irrigated land | Other agriculture |
| Vineyards | Other agriculture |
| Fruit trees and berry plantations | Other agriculture |
| Olive groves | Other agriculture |
| Pastures | Other agriculture |
| Annual crops associated with permanent crops | Other agriculture |
| Complex cultivation patterns | Other agriculture |
| Land principally occupied by agriculture, with significant areas of natural vegetation | Other agriculture |
| Agro-forestry areas | Other agriculture |
| Road and rail networks and associated land | OtherUrban |
| Airports | OtherUrban |
| Mineral extraction sites | OtherUrban |
| Construction sites | OtherUrban |
| Port areas | Port |
| Rice fields | Ricefields |
| Salines | Salines |
| Sport and leisure facilities | Sport Facilities |


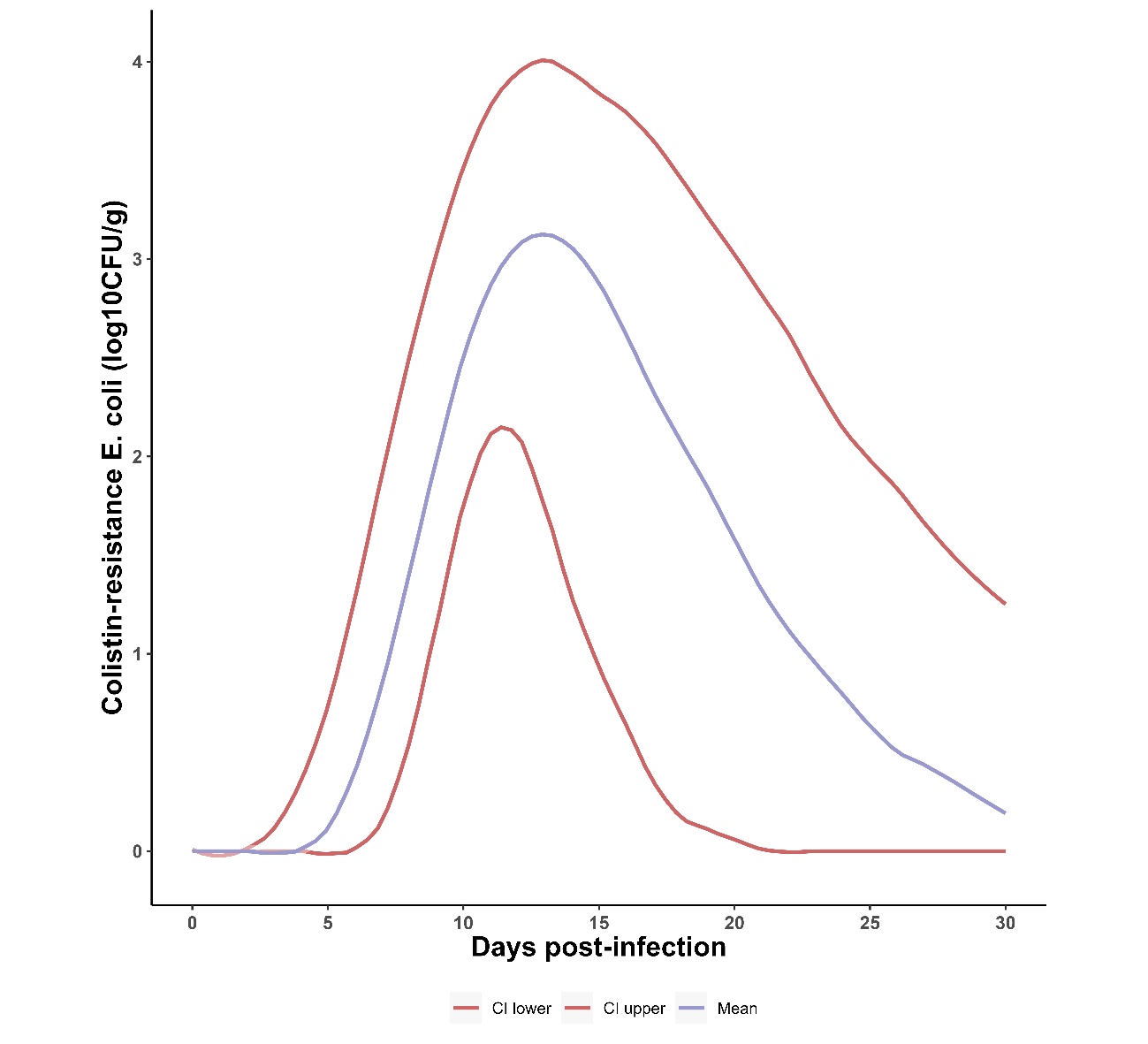


**Figure S1**. Lognormal curve derived from Franklin et al. (2020) showing the relationship of Colony Formation Unit (CFU) concentration with shedding time during 30 days. Mean and 95% CI are shown.


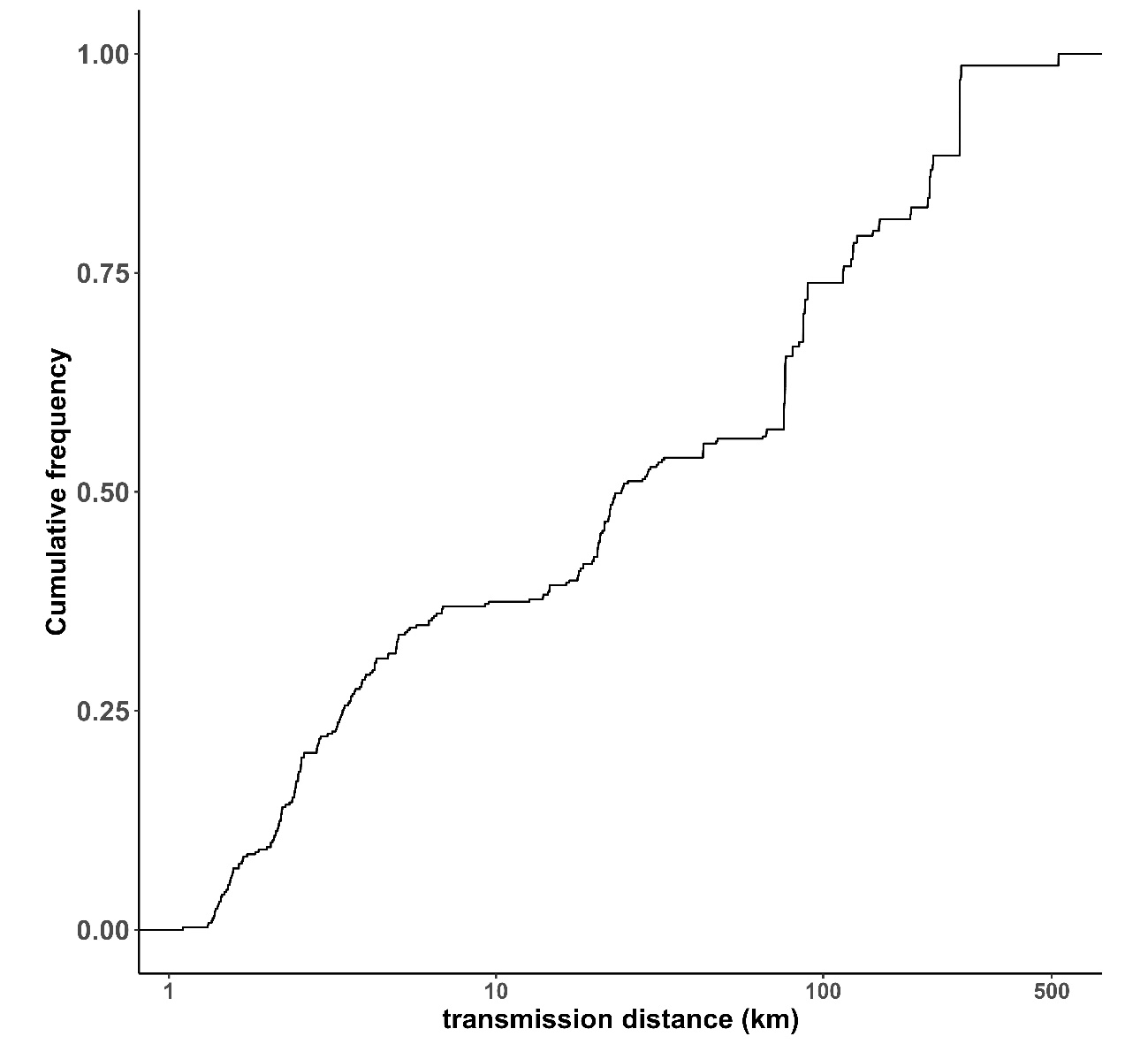


**Figure S2.** Cumulative frequency of potential ARB dispersal distances of black headed gull individuals over a shedding period of 30 days. The value of 1 in y-axis means that all trajectories are included by that distance (i.e. the point reached at 521 km).
